# Rapid intravenous rehydration of children with acute gastroenteritis and dehydration: a systematic review and meta-analysis

**DOI:** 10.1186/s12887-018-1006-1

**Published:** 2018-02-09

**Authors:** M. A. Iro, T. Sell, N. Brown, K. Maitland

**Affiliations:** 1 Oxford Vaccine Group, Department of Paediatrics and the NIHR Biomedical Research Centre, University of Oxford, Headington, Oxford, OX3 7LE UK; 2 Department of Paediatrics, Salisbury District Hospital, Salisbury, SP2 8BJ UK; 3 Department of Child Health, Aga Khan University, Karachi, Pakistan; 4 Department of Paediatrics, Faculty of Medicine, Wellcome Trust Centre for Clinical Tropical Medicine, Imperial College, W2 1PG, London, UK; 5 Clinical Trials Facility, KEMRI Wellcome Trust Research Programme, PO Box 230, Kilifi, Kenya

**Keywords:** Acute gastroenteritis, Dehydration, Intravenous rehydration, Systematic review, Emergency care, Africa, Asia

## Abstract

**Background:**

The World Health Organization (WHO) recommends rapid intravenous rehydration, using fluid volumes of 70-100mls/kg over 3–6 h, with some of the initial volume given rapidly as initial fluid boluses to treat hypovolaemic shock for children with acute gastroenteritis (AGE) and severe dehydration. The evidence supporting the safety and efficacy of rapid versus slower rehydration remains uncertain.

**Methods:**

We conducted a systematic review of randomised controlled trials (RCTs) on 11th of May 2017 comparing different rates of intravenous fluid therapy in children with AGE and moderate or severe dehydration, using standard search terms. Two authors independently assessed trial quality and extracted data. Non-RCTs and non-English articles were excluded. The primary endpoint was mortality and secondary endpoints included adverse events (safety) and treatment efficacy.

**Main results:**

Of the 1390 studies initially identified, 18 were assessed for eligibility. Of these, 3 studies (*n* = 464) fulfilled a priori criteria for inclusion; most studied children with moderate dehydration and none were conducted in resource-poor settings. Volumes and rates of fluid replacement varied from 20 to 60 ml/kg given over 1-2 h (fast) versus 2-4 h (slow). There was substantial heterogeneity in methodology between the studies with only one adjudicated to be of high quality. There were no deaths in any study. Safety endpoints only identified oedema (*n* = 6) and dysnatraemia (*n* = 2). Pooled analysis showed no significant difference between the rapid and slow intravenous rehydration groups for the proportion of treatment failures (*N* = 468): pooled RR 1.30 (95% CI: 0.87, 1.93) and the readmission rates (*N* = 439): pooled RR 1.39 (95% CI: 0.68, 2.85).

**Conclusions:**

Despite wide implementation of WHO Plan C guideline for severe AGE, we found no clinical evaluation in resource-limited settings, and only limited evaluation of the rate and volume of rehydration in other parts of the world. Recent concerns over aggressive fluid expansion warrants further research to inform guidelines on rates of intravenous rehydration therapy for severe AGE.

**Electronic supplementary material:**

The online version of this article (10.1186/s12887-018-1006-1) contains supplementary material, which is available to authorized users.

## Background

The global health burden of acute gastroenteritis (AGE) is substantial. Worldwide, 1·73 billion episodes of diarrhoea (of which 36 million progressed to severe episodes) were reported in 2010 in children under 5 years [1]. In this age group, AGE is the single largest cause of mortality after acute respiratory illnesses resulting in approximately 700, 000 deaths annually year [1], the vast majority occurring in sub-Saharan Africa and South Asia [2, 3]. Preventative measures including clean drinking water and improvement in sanitation and rotavirus vaccination have led to some decrease in the incidence. In addition, there has been a modest improvement in case management and outcomes in resource-limited settings as a result of the use of oral rehydration therapy and the introduction of adjunctive oral zinc treatment to standard management [4, 5]. Nevertheless, mortality from AGE remains unacceptably high. A recent large case-control study of moderate to severe acute gastroenteritis conducted in four centres in Africa and three centres in Asia (Global Enteric Multicentre study ‘GEMS’) showed that the odds of dying during a 90-day follow-up period was 8·5-fold higher in patients with moderate-to-severe AGE than in non-AGE controls [3]. One quarter of fatalities occurred within 7 days during the primary diarrhoeal health care encounter indicating that current management recommendations warrant re-appraisal.

For children with moderate to severe AGE, rehydration with oral or intravenous isotonic fluids to correct fluid and electrolyte deficits and on-going losses is the mainstay of treatment. Where oral rehydration is not feasible, rehydration by nasogastric tube is the preferred option and recommended before intravenous rehydration in some guidelines [6]. However, some aspects of current management recommendations are controversial [7]. First, the decision to treat with either oral or intravenous fluids is largely based upon an assessment of level of dehydration, which has been shown to be notoriously unreliable [8, 9]. Second, though the evidence supporting the recommendations are scanty, current World Health Organization (WHO) guidelines for the treatment of severe dehydration (‘Plan C’) are based on rapid, intravenous administration of isotonic fluids. Plan C recommends a minimum of 100mls/kg, the equivalent volume replacement for losses in those with 10% dehydration. Plan C is given in two stages over 3–6 h, dependent on age. Recommending an initial 30 ml/kg to be administrated rapidly (30–60 min) and the remaining 70 ml/kg more slowly over 2.5–5 h respecitvely in infants aged under 12 months and older [10]. For those with hypovolaemic shock initial management recommends fluid boluses (of up to 60 ml/kg) rehydration for children which is followed by step 2 of plan C (70 ml/kg more slowly over 2.5–5 h). Whilst the aggressive regime may be appropriate for cholera, the only infective cause of secretory-diarrhoea (leading to excess fluid and electrolyte loss), it may not be generalisable to non-cholera AGE, the dominant cause of AGE worldwide [3]. Despite lack of formal testing, the WHO expert review group indicated this as a strong recommendation [11].

There are, however, increasing concerns over the safety of rapid intravenous correction of fluid deficits. The only controlled trial (FEAST) assessing fluid bolus therapy in children with presumed sepsis in sub-Saharan Africa demonstrated a significantly higher mortality in those children receiving fluid-bolus therapy [12], including a large group with severe dehydration [13]. Although the FEAST trial raises important questions about the safety of rapid intravenous fluid therapy in severe febrile illnesses, children with severe dehydration secondary to AGE were not included in the trial, therefore it is uncertain whether rapid fluid replacement to correct dehydration, the cornerstone of standard management in this condition, is safe. We therefore conducted a systematic review of the evidence underpinning current guidelines for intravenous rehydration.

### Objectives

To conduct a critical appraisal of available evidence on the safety and efficacy of the rapidity of intravenous fluid therapy for the correction of moderate-severe dehydration in children with AGE.

## Methods

We did not publish a protocol prior to conducting this review. We registered our search strategies on PROSPERO on 21st May 2017 (review number 67532). We used pre-defined rules relating to eligibility criteria, information sources to be searched, study selection, data collection process, and assessment of risk of bias in identified studies.

### Selection criteria and process

#### Population

Children aged 0 to 18 years with a diagnosis of acute gastroenteritis and moderate or severe dehydration. Due to the possible variation in the diagnosis of acute gastroenteritis, where this was not clearly defined in the manuscript, we planned to use either the ESPGHAN [6] or WHO [10] definition for acute gastroenteritis. Where the level of dehydration in trial participants was not clearly defined we used the WHO guideline definition for moderate and severe dehydration [6]. We excluded studies with severe malnutrition and chronic or persistent diarrhoea according to WHO definitions (i.e. lasting ≥14 days).

### Intervention

Interventions included any form of intravenous rehydration with isotonic solutions e.g. 0.9% sodium chloride or Ringer’s lactate for rehydration. Studies that involved the use of hypotonic solutions were excluded since these are not recommended for intravenous rehydration.

### Comparison

A comparator was considered as any of the above intravenous isotonic solution given at different rates for rehydration. Enteral therapies were not considered.

### Outcomes

Our primary outcome of interest was mortality. Secondary endpoints included treatment efficacy (as defined in the study protocols - see below) and safety outcomes (within 28 days of rehydration). These included:

- Proportion of participants with a pre-defined serious adverse event (other than death). Where this was not clear we used the definition from the International Conference on Harmonisation (ICH) Harmonised Tripartite Guideline [14]: any adverse event is life-threatening, or requires hospitalisation or prolongation of hospital stay, results in persistent or significant disability/incapacity. Pre-specified SAEs were new onset seizures, pulmonary oedema, cerebral oedema and cardiac failure.
- Proportion of participants with dysnatraemia at 4–6 h after the initiation of intravenous rehydration. Dysnatraemia was defined as a serum sodium level outside the normal range (135-145 mmol/L) [15]. Where dysnatraemia was present at enrolment, we planned to compare the magnitude (percentage) of decrease or increase in serum sodium levels from baseline at enrolment.

### Efficacy (secondary) outcomes

- Time to successful rehydration as continuous or categorised data. Any definition of ‘successful rehydration’ pre-specified in the manuscript based on clinical parameters such as (but not restricted to) weight gain, improved urinary output, increased skin turgor, improved level of consciousness and able to keep down oral fluids was acceptable.
- Length of hospital stay either as continuous or categorised data.
- Mean duration of diarrhoea in hours or days, as continuous or categorised data.
- Treatment failure using any criteria defined in the manuscript.

### Study type

We included only randomised controlled trials (RCTs).

### Search methods for identification of studies

#### Electronic searches

A comprehensive literature search (Additional file 1: Table S1a and S1b) of the following databases was conducted on the 11th of May 2017 using a search strategy developed by a research librarian:

- Ovid MEDLINE ® In-Process & Other Non-Indexed Citations and Ovid MEDLINE (1946 – May 11, 2014)
- Embase (1974 – May 2017)
- Global Health (1973 – May 2017)
- Global Health Library (Virtual Health Library)
- Science Citation Index Expanded (SCI-EXPANDED); and Conference Proceedings Citation Index-Science (CPCI-S) (Web of Science) (1945 – May 2017)
- Cumulative Index to Nursing and Allied Health Literature (CINAHL)(1982 – May 2017)
- Cochrane Database of Systematic Reviews (Issue 5 of 12, May 2017)
- Cochrane Central Register of Controlled Trials (Issue 4 of 12, April 2017)
- The Database of Abstracts of Reviews of Effects (Issue 2 of 4, April 2015)
- ClinicalTrials.gov (http://clinicaltrials.gov) (last accessed in May 2017).
- The World Health Organization (WHO) International Clinical Trials Registry Portal (ICTRP) search portal (http://apps.who.int/trialsearch/)

We performed a visual scan of reference lists of relevant studies and a Google search for additional studies. We limited our search to trials published in English language. No restriction was placed on year of publication.

### Selection of studies

Two reviewers (MI, TS) independently screened the results of the literature search and assessed the eligibility of studies to be included. Level 1 screening involved a broad screen of study titles and abstracts. Level 2 screening entailed a comprehensive assessment of the full text of studies that meet the inclusion criteria, or in cases where a definite decision could not be made based on the title and/or abstract alone. We compared multiple reports of the same study, and selected the most comprehensive report. Duplicates were excluded. Relevant data relating to the Population, Intervention, Comparison, Outcome, Study design (PICOS) criteria were extracted using a pre-agreed data extraction sheet.

### Assessment of bias in the included studies

The reviewers (MI and TS) assessed the risk bias of each randomised controlled trial using ‘The Cochrane Collaboration’s tool for assessing the risk of bias [16] to evaluate internal validity in terms of: i) selection (sequence generation and allocation concealment); (ii) performance and detection (blinding of participants, personnel, and outcome assessors); (iii) attrition (incomplete outcome data) and (iv) reporting (selective outcome reporting). We used the summary quality assessment at the analysis stage to interpret the results. For each domain and for the summary a, we assigned the risk of bias categories as: (i) ‘low risk’; (ii) ‘unclear risk’ and (iii) ‘high risk’ [17]. We rated a study as being of good methodological quality when the level of bias was low in all four domains, or of lower quality when the level of bias was high in at least one of the four domains.

### Assessment of heterogeneity

We assessed statistical heterogeneity by visually inspecting Forrest plots and using the chi-squared test for heterogeneity (with a *P* value < 0.10 for significance) and the I^2^ statistic as a measure of inconsistency across studies [16].

### Data synthesis

We aimed to generate pooled estimates using a fixed-effect model meta-analysis where trials were judged to be sufficiently statistically homogenous (I^2^ < 50%) and a random-effects model where we found significant heterogeneity (I^2^ > 50%).

### Statistical analysis and summary measures

We carried out statistical analyses using Review Manager 2014. For dichotomous data, we report on relative risk (RR) with 95% confidence intervals (CIs). For continuous data, we planned to use weighted mean difference (WMD).

## Results

### Study selection

**Fig. 1:**
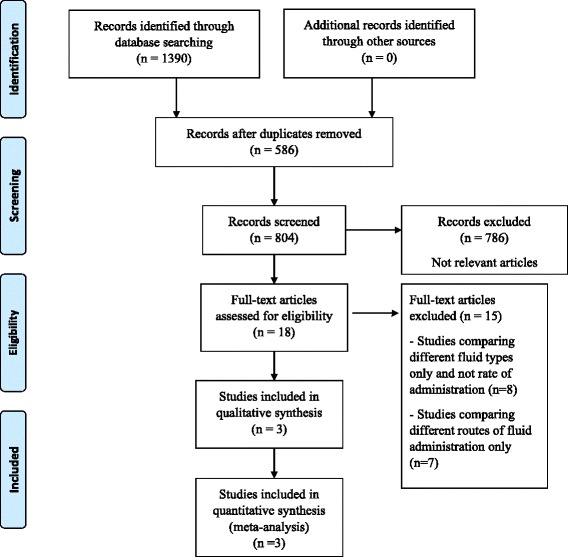
Flow diagram for selection of randomised trials and reasons for study exclusion. Footnote CRCT = Cochrane Register of Clinical Trials

The process of study identification was shown in Fig. 1. The search identified 1390 studies − 1155 and 235 in the initial and updated search respectively (Additional file 1: Table S1a and S1b). Of the total number identified, 586 were duplicates and therefore were excluded. A total of 786 articles were excluded at Level 1 screening since the title and/or abstracts did not suggest that the report related to a trial of rapid intravenous rehydration in children with acute gastroenteritis. We identified 18 RCTS involving intravenous treatment in children with AGE and reviewed the full text of each. Only 3 studies were eligible to be included in this review.

### Included studies

All the eligible RCTs [18–20] were identified through the database search. Two of the included studies [18, 19] were conducted in resource-rich countries. Canada and the USA respectively. While the third [20] was conducted in Iran. The characteristics of the included studies are summarised in Table 1. Freedman’s study was conducted in a Paediatric ED in Toronto and enrolled children from 3 to 11 months who were dehydrated as a result of AGE and in whom oral rehydration had not been feasible. Outcomes were defined by a validated dehydration score. There were no protocol deviations noted. Nager enrolled children from 3 to 36 months in Los Angeles and randomised to ‘ultra rapid’ 50 ml/kg rehydration over 1 h (intervention) or ‘standard’ /control 50 ml/kg over 3 h. There was a 95% completion rate. Azarfar enrolled children in Tabriz, Iran with gastroenteritis unable to tolerate oral fluids. They were randomised to 20–30 ml/kg over 2 h (intervention) or 24 h (control) and primarily compared by proportion in whom vomiting ceased.

**Table 1:** Characteristics of included studies

Freedman 2011 [18]	
Methods	Randomised controlled trial conducted in the emergency department of the Hospital for Sick Children, Toronto, Canada. Study period between December 2006 and April 2010
Study aim	To determine if rapid rather than standard intravenous rehydration results in improved hydration and clinical outcomes when administered to children with gastroenteritis.
Participants	Inclusion criteria: Age > 90 days; diagnosis of dehydration secondary to gastroenteritis and refractory to oral rehydration.
	Exclusion criteria: children weighing < 5 kg or > 33 kg, requiring for fluid restriction, had a suspected surgical condition, had a history of a severe chronic systemic disease, abdominal surgery, or bilious vomit, had hypotension, hypoglycaemia or hyperglycaemia, insurmountable language barrier or lack of telephone for follow up call.
Interventions	One hundred and twelve infants received 60 mL/kg of 0.9% saline over 60 min (rapid rehydration) and 114 children received 20 mL/kg over 60 min (standard rehydration).
Allocation	1:1
Outcomes	Primary: Rehydration defined as a score on the clinical dehydration scale of ≤1 two hours after the start of treatment.
	Secondary: Prolonged treatment – a composite measure defined as admission to an inpatient unit at the index visit or admission within 72 h of randomisation or a stay in the emergency department longer than 6 h after the start of treatment; score on a clinical dehydration scale; adequate oral fluid intake defined as consuming at least 5 mL/kg of liquid per 2 h time period; time to discharge defined as time between start of treatment and discharge from the emergency department of inpatient unit; repeat emergency department visit within 72 h; and attending physician’s comfort with discharge at two and four hours, reported on a 5-point Likert scale.
Nager 2008 [19]	
Methods	Pilot randomised controlled convenience sample study in the emergency department of the Children Hospital in Los Angeles, USA
Study aim	To provide some evidence for our belief that the ultra protocol could be performed effectively with similar results as the standard hydrating method.
Participants	Ninety-two children aged 3 to 36 months
	Inclusion criteria: acute (< 7 days) complaints of vomiting and/or diarrhoea) and moderate dehydration and failure of oral rehydration.
	Exclusion criteria: severe dehydration, shock, suspected intussusception, appendicitis, mal-rotation, recent trauma, meningitis, or congestive heart failure or if any of these diagnoses appeared as the study progressed; chronic disease or significant laboratory abnormality including Na < 130 or > 150 mmol/L and/or K < 3.2 or > 5.5 mmol/L.
Interventions	50 mL/kg of normal saline IV administered for 1 h (ultra rapid IV hydration) or 50 mL/kg normal saline IV for 3 h (standard hydration)
Allocation	1:1
Outcomes	Efficacy of treatment by assessing Success and timing of rehydration, study failures (defined as requirement for admission), output (urine, emesis, stool) during the treatment phase, pre- and post treatment laboratory abnormalities, number of return visits, and whether serious complications occurred.
Azarfar 2014 [20]	
Methods	Randomised controlled trial conducted in the emergency department in a tertiary centre (Tabriz children’s hospital) in Tabriz, North-West of Iran.
Objective	To evaluate the effect of rapid intra- venous rehydration to resolve vomiting in children with acute gastroenteritis.
Participants	Inclusion criteria: 150 Children with moderate dehydration or vomiting due to gastroenteritis who had not responded to oral rehydration therapy.
	Exclusion criteria: severe dehydration, shock, and hypotension, electrolyte abnormalities, none or mild dehydration.
Intervention	20-30 mL/kg of a crystalloid solution over either 2 h (intervention group) or 24 h (control group).
Allocation	1:1
Outcomes	Primary outcome: Resolution of vomiting in children receiving rapid intravenous rehydration.
	No secondary outcomes.

### Excluded studies

Fifteen studies of intravenous rehydration in AGE were excluded (Additional file 1: Table S2). Seven of these compared different routes of fluid administration (enteral versus parenteral) while eight compared different types of intravenous fluids not rates of fluid administration, and therefore failed to meet our eligibility criteria. One unpublished study of slow versus rapid rehydration in severely malnourished children was identified through Clinicaltrials.gov (NCT02216708) but failed to meet our eligibility criteria. No studies were excluded because the trial participants did not meet the ESPGHAN/WHO definitions for acute gastroenteritis or the WHO definition for moderate or severe dehydration.

### Risk of bias within studies

**Fig. 2:**
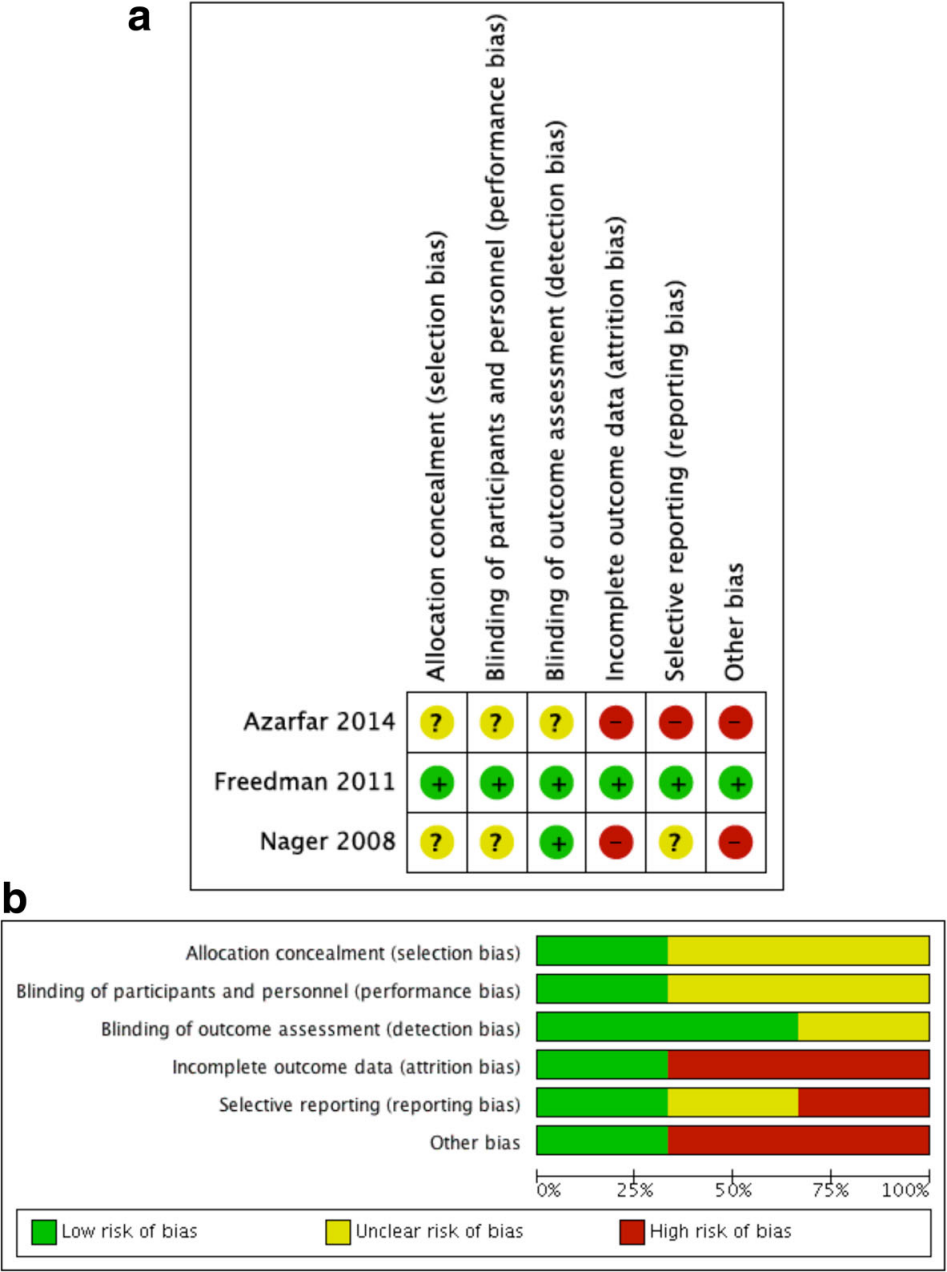
**a** Risk of bias summary: authors’ judgements for each included study. **b** Risk of bias graph: authors’ judgements presented as percentages for all studies

Only one study [18] was rated as having low risk of bias while the other two studies [19, 20] were rated as being of lower methodological quality. The risk of bias assessment for all 3 included studies is shown in Fig. 2a and b and Additional file 1: Table S3.

### Results of included studies

There was significant heterogeneity in the design and outcomes measured in all 3 studies. We report on the results of individual studies and these are organised using the pre-specified primary and secondary outcomes for this review.

- Primary outcome.

There were no deaths in any of the studies.

(b) Secondary, safety outcomes

We found no reports of any of our pre-specified safety outcomes of interest (new onset seizures, pulmonary oedema, cerebral oedema and cardiac failure). Following 4 h of fluid replacement [18] (*n* = 226) found that serum sodium levels were similar for both groups (rapid versus standard): 138 mmol/l (2.0) vs. 137.5 (2.0); *p* = 0.06 with only one child per group developing a decrease in serum sodium concentration. The magnitude of this decrease was 5.8% (138 mmol/L to 130 mmol/L) in the rapid rehydration group compared to 1.5% decline (130 mmol/L to 128 mmol/L) in the standard rehydration group. In addition, 1/114 (0.9%) vs. 1/112 (0.9%) (rapid versus standard rehydration group) developed an interstitial displacement of the intravenous catheter resulting in extravasation [18].

### (b) Efficacy, secondary outcomes

#### (i) Pre-specified efficacy outcomes of interest reported

**Freedman **[18]: 41/114 (36%) of children (rapid rehydration group) versus 33/112 (29%) in the standard rehydration group were considered as rehydrated at 2 h after commencement of rehydration therapy (absolute difference for rapid vs. standard 6.5%, 95% CI − 5.7% to 18.7%; *p* = 0.32). Prolongation of treatment was reported in 59/114 (52%) of the rapid rehydration group and 48/112 (43%) in the standard group (absolute difference for rapid vs. standard, 8.9%, 21.0% to − 5.0%; *P* = 0.19). More children in the rapid intravenous rehydration group were admitted to hospital at the index visit (33 vs. 19, *p* = 0.04) with this difference persisting following exclusion of children admitted to hospital because of their metabolic acidosis [number needed to harm = 9, 95% CI (4 to 57)].

**Nager **[19]: Treatment failure necessitating admission was reported in 1/46 (2%) of children of the ultra-rapid rehydration group versus 3/46 (6.5%) in the standard group. Overall, 13/88 (14.8%) of 88 subjects returned following discharge: 7/45 (15.6%) ultra-rapid (CI, 6.5%–29.5%) and 6/43 (14.0%) standard (CI, 5.3%–28.0%), *p* = 0.999.

**Azarfar **[20]: At two hours following commencement of intravenous fluid therapy, 63/75 (84%) of children (rapid rehydration group) versus 62/75 (82%) in the standard group were considered as rehydrated or had resolved their vomiting and were thus discharged (*p* ≥ 0.05). Two subjects in the intervention group and none in the standard group required readmission following discharge.

None of the included studies reported on mean duration of diarrhoea as an outcome measure.

#### (ii) Efficacy outcomes reported in the included studies but not pre-specified in this review

**Freedman **[18]: Change in serum bicarbonate levels (standard deviation) before and after treatment (standard vs. rapid group) was 0.56 (1.9) vs. -0.31(2.2) mmol/L, *p* = 0.01. There were no significant differences in (i) mean dehydration score (ii) proportion rehydrated at 4 h, (iii) adequacy of oral intake at 2 and 4 h, (iv) physician comfort with discharge at 2 and 4 h.

**Nager **[19]: There was no significant difference in heart rate decrease (*p* = 0.163), weight gain (*p* = 0.343), and mean laboratory values of serum potassium, glucose, blood urea nitrogen, creatinine and CO2 measured pre and post rehydration.

**Azarfar **[20]: None.

### Meta-analysis

**Fig. 3:**
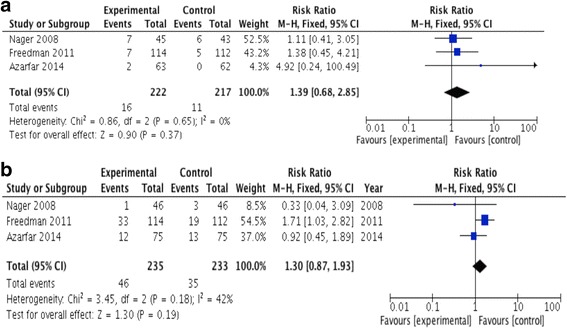
**a** Forest Plot: Treatment failure requiring admission during initial visit: rapid/ultra-rapid (experimental) versus slow/standard (control) intravenous rehydration. **b** Readmission following initial discharge: rapid/ultrarapid (experimental) versus slow/standard (control) intravenous rehydration

For the primary outcome (mortality) and pre-specified safety endpoint there were no events reported. Heterogeneity precluded meta-analysis of the safety endpoints. The efficacy outcomes were sufficiently homogenous for pooled analysis and showed no significant difference in the proportion of treatment failures in the rapid versus slow rehydration groups (*N* = 468): RR 1.30 (95% CI: 0.87, 1.93) (Fig. 3a) Similarly, there was no difference in the readmission rate for both groups (*N* = 439): RR 1.39 (95% C: 0.68, 2.85) (Fig. 3b). Only one study [18] reported time to resolution of dehydration, finding no significant difference between treatment assignment and successful rehydration by two hours (odds ratio 1.8 (95% CI 0.90–3.5); *p* = 0.10).

None of the studies reported on length of hospital stay or mean duration of diarrhoea.

## Discussion

Data from this review reveals a paucity of clinical trials to support a robust evidence for the use of rapid intravenous rehydration in children with moderate to severe dehydration due to AGE. We identified only three studies meeting our pre-defined criteria. Only one study included children with severe dehydration while the other two studies included only children with moderate dehydration. None of the studies evaluated the WHO Plan C rapid rehydration guideline, recommended for the management of severe dehydration, and none were conducted in resource-limited settings. Each used a different methodology, rate of rehydration and tools of assessment. Albeit heterogenous, the meta-analysis of these trials did not suggest superiority of rapid or ultra-rapid over slower rehydration. Moreover, the relatively small number of patients included in the published, single-centred trials with endpoints primarily focusing on non-critical indicators of treatment efficacy in the absence of mortality endpoints limit the generalisability to resource-poor settings, where mortality remains an important outcome in this condition.

The available data either informing or evaluating current treatment guidelines present a number of limitations. First only one trial [18] was sufficiently powered to detect any treatment effects. In that trial the estimated sample size provided 80% power to detect a 20% point difference between in the proportion of children rehydrated after two hours of commencing rehydration treatment [18]. Second, the low quality of the two other trials [19, 20] precludes conclusions pertaining to safety and efficacy of current guidelines. Finally, all published trials were conducted in well-resourced emergency rooms so it is unclear whether the findings would be applicable to low and middle income countries (LMIC) which face the bulk of the global burden of AGE.

The 2013 version of the WHO pocket book [10] contained no amendments to the original guidelines, and, as a result, recommendations for fluid management of AGE are now over a decade old. Notable it that the guidance remains “strongly recommended’ though the evidence base is reported as weak [11, 12].

The FEAST trial, conducted in sub-Saharan Africa, demonstrated that children randomised to fluid boluses of either saline or albumin had a 3.3% higher mortality than children receiving only maintenance fluids [12]. A subsequent sub-analysis showed that whilst there was evidence of improved short-term hemodynamic effects with bolus, this did not result in a better outcome with the excess mortality was due to cardiogenic or shock as terminal clinical events (*n* = 123; 4.6% in bolus versus 2.6% in control, *P* = 0.008) rather than respiratory or neurological terminal clinical events as anticipated [13]. The trial re-emphasises the importance of testing all recommendations in which the evidence base is weak. Relevant to this review is that children with AGE were not enrolled in FEAST, thus the results cannot be extrapolated and therefore further research is required to clarify whether these findings are also relevant to those with diarrhoea-related dehydration. A large prospective multicentre observational study in Kenya examining physcians use of resusciation and rehydration fluids with respect to outcome iincluded a large subgroup with severe dehydrating diarrhoea [21]. Most fluid boluses given for resuscitation of hypovolaemic shock secondary to dehydration/diarrhoea (94%, 582/622), and case fatality was high in this group (34%, 211/622). Overall mortality was 7.9% (798/10,096) in children with dehydration/diarrhoea [21].

Though there are some physiological differences between the two illness phenotypes with intracellular dehydration the first pathological step in AGE, shock and electrolyte disturbance are common to both. This suggests, at the very least, that the current WHO guidance of rapid rehydration should be formally assessed through large phase III RCT with disability-free mortality as the primary endpoint in an appropriate resource limited setting. A safety and pilot efficacy study (using physiological surrogates of efficacy) has been registered on ISRCTN 67518332 aiming to compare the current WHO Plan ‘C’ rehydration protocol with a strategy that aims to give a slower rehydration regimen (without fluid bolus) using the same total volume (100 ml/kg of Ringers Lactate) over 8 h, irrespective of age. The hypothesis indicates that slower rehydration is equally effective in rehydration but is associated with fewer fluid related adverse effects with a view to informing the design of a future definitive multi-site Phase III trial.

The strengths of the review include a rigorous search and reporting according to the established PRISMA guidelines. Potential weaknesses include the possibility of undetected unpublished work, non-inclusion of non-English studies and the heterogeneity in identified studies precluding a formal meta-analysis to augment the systematic review. Another review has recently been published with similar conclusions for management of AGE in emergency rooms in high income (HMIC) settings [22]. However, our review sought to address the global burden of disease and treatment challenges for AGE rehydration and, given the paucity of data, we can only conclude that robust trials are long overdue.

## Conclusion

There is no high quality trial evidence from LMIC to support the current WHO guidance of rapid intravenous rehydration in children with acute gastroenteritis complicated by severe dehydration, nor is there relevant evidence from trials in well-resourced settings that demonstrated a favourable benefit of rapid rehydration over slow rehydration. We suggest this dilemma can only be robustly addressed in future by an adequately powered randomised trial.

## Additional file

Additional file 1: **sTable 1a.** Search results (up to October 2014). **sTable 1b.** Search results (October 2014 to May 2017). **sTable 2.** Excluded studies. **sTable 3.** Risk of bias for included studies. (DOCX 145 kb)

## Abbreviations

AGE: Acute gastroenteritis
CIs: Confidence intervals
ED: Emergency department
ESPGHAN: European Society for Pediatric Gastroenterology, Hepatology, and Nutrition
FEAST: Fluid as a supportive therapy
LMIC: Low and middle-income countries
RR: Relative risk
WHO: World Health Organization
