# Supplementary material for: Rapid intravenous rehydration of children with acute gastroenteritis and dehydration: a systematic review and meta-analysis
Source: BMC Pediatr. 2018 Feb 9;18:44. doi: 10.1186/s12887-018-1006-1 (PMC5807758; doi:10.1186/s12887-018-1006-1)
Supplement: Supplementary file 1 — sTable 1a. Search results (up to October 2014). sTable 1b. Search results (October 2014 to May 2017). sTable 2. Excluded studies. sTable 3. Risk of bias for included studies. (DOCX 145 kb) [file 12887_2018_1006_MOESM1_ESM.docx]

**Supplementary Table 1: SEARCH RESULTS (Up to October 2014)**

**MEDLINE SEARCH**

| # ▼ | Searches | Results |
| --- | --- | --- |
| 1 | adolescent/ or exp child/ or infant/ or infant, newborn/ | 2957714 |
| 2 | (child* or adolescen* or teen* or infan* or boy? or girl? or pediatric* or paediatric*).ti,ab. | 1560297 |
| 3 | 1 or 2 | 3302323 |
| 4 | exp diarrhea/ or vomiting/ or gastroenteritis/ or enteritis/ | 80234 |
| 5 | (diarrh* or vomit*or gastroenteritis or enteritis).ti,ab. | 90127 |
| 6 | Dehydration/ | 10790 |
| 7 | dehydrat*.ti,ab. | 32066 |
| 8 | 4 or 5 or 6 or 7 | 166221 |
| 9 | administration, intravenous/ or infusions, intravenous/ | 50234 |
| 10 | (intravenous or intra-venous or iv).ti,ab. | 520464 |
| 11 | 9 or 10 | 543083 |
| 12 | exp Fluid Therapy/ | 15109 |
| 13 | Rehydration Solutions/ | 1267 |
| 14 | Sodium Chloride/ | 50879 |
| 15 | 12 or 13 or 14 | 65798 |
| 16 | 11 and 15 | 5564 |
| 17 | ((intravenous or intra-venous or iv) adj10 (rehydrat* or hydrat* or fluid* or saline or solution*)).ti,ab. | 22243 |
| 18 | 16 or 17 | 24888 |
| 19 | (rapid* or ultrarapid* or ultra-rapid*).ti,ab. | 730039 |
| 20 | Time Factors/ | 1041221 |
| 21 | 19 or 20 | 1708872 |
| 22 | 18 and 21 | 4048 |
| 23 | ((fast* or time? or timing or hour? or minute?) adj10 (rehydrat* or hydrat* or fluid* or saline or solution*)).ti,ab. | 59009 |
| 24 | 11 and 23 | 6393 |
| 25 | ((rapid* or ultrarapid* or ultra-rapid* or fast* or time? or timing or hour? or minute?) adj5 (rehydrat* or hydrat* or fluid* or saline or solution*)).ti,ab. | 33926 |
| 26 | ((rapid* or ultrarapid* or ultra-rapid* or fast* or time? or timing or hour? or minute?) and (rehydrat* or hydrat* or fluid* or saline or solution*)).ti. | 4163 |
| 27 | riv.ti,ab. | 137 |
| 28 | 22 or 24 or 25 or 26 or 27 | 42341 |
| 29 | 3 and 8 and 28 | 520 |
| 30 | limit 29 to "reviews (maximizes specificity)" | 16 |
| 31 | randomized controlled trial.pt. | 398164 |
| 32 | controlled clinical trial.pt. | 90534 |
| 33 | randomized.ab. | 317365 |
| 34 | placebo.ab. | 163089 |
| 35 | drug therapy.fs. | 1777970 |
| 36 | randomly.ab. | 227529 |
| 37 | trial.ab. | 331133 |
| 38 | groups.ab. | 1431004 |
| 39 | 31 or 32 or 33 or 34 or 35 or 36 or 37 or 38 | 3513892 |
| 40 | exp animals/ not humans.sh. | 4082112 |
| 41 | 39 not 40 | 3017938 |
| 42 | 29 and 41 | 248 |
| 43 | exp cohort studies/ | 1428666 |
| 44 | cohort$.tw. | 309003 |
| 45 | controlled clinical trial.pt. | 90534 |
| 46 | epidemiologic methods/ | 30762 |
| 47 | limit 46 to yr="1971 - 1988" | 9584 |
| 48 | 43 or 44 or 45 or 47 | 1640141 |
| 49 | 29 and 48 | 89 |

**EMBASE SEARCH**

| # ▼ | Searches | Results |
| --- | --- | --- |
| 1 | child/ or boy/ or girl/ or infant/ or preschool child/ or school child/ or adolescent/ | 2403080 |
| 2 | (child* or adolescen* or teen* or infan* or boy? or girl? or pediatric* or paediatric*).ti,ab. | 1856885 |
| 3 | 1 or 2 | 3009101 |
| 4 | diarrhea/ or acute diarrhea/ or infantile diarrhea/ or vomiting/ or gastroenteritis/ or enteritis/ or acute gastroenteritis/ or gastroenteritis/ or infantile gastroenteritis/ | 313929 |
| 5 | (diarrh* or vomit*or gastroenteritis or enteritis).ti,ab. | 113274 |
| 6 | dehydration/ | 27938 |
| 7 | dehydrat*.ti,ab. | 37503 |
| 8 | 4 or 5 or 6 or 7 | 391788 |
| 9 | intravenous drug administration/ | 356319 |
| 10 | (intravenous or intra-venous or iv).ti,ab. | 646242 |
| 11 | 9 or 10 | 890236 |
| 12 | fluid therapy/ | 15280 |
| 13 | rehydration/ | 4496 |
| 14 | 12 or 13 | 19582 |
| 15 | 11 and 14 | 3074 |
| 16 | sodium chloride/iv | 1291 |
| 17 | infusion fluid/ | 8176 |
| 18 | ((intravenous or intra-venous or iv) adj10 (rehydrat* or hydrat* or fluid* or saline or solution*)).ti,ab. | 27894 |
| 19 | 15 or 16 or 17 or 18 | 35771 |
| 20 | (rapid* or ultrarapid* or ultra-rapid*).ti,ab. | 826773 |
| 21 | Time/ | 371322 |
| 22 | 20 or 21 | 1182403 |
| 23 | 19 and 22 | 3301 |
| 24 | ((fast* or time? or timing or hour? or minute?) adj10 (rehydrat* or hydrat* or fluid* or saline or solution*)).ti,ab. | 70698 |
| 25 | 11 and 24 | 8765 |
| 26 | ((rapid* or ultrarapid* or ultra-rapid* or fast* or time? or timing or hour? or minute?) adj5 (rehydrat* or hydrat* or fluid* or saline or solution*)).ti,ab. | 40245 |
| 27 | ((rapid* or ultrarapid* or ultra-rapid* or fast* or time? or timing or hour? or minute?) and (rehydrat* or hydrat* or fluid* or saline or solution*)).ti. | 4463 |
| 28 | riv.ti,ab. | 181 |
| 29 | 23 or 25 or 26 or 27 or 28 | 49435 |
| 30 | 3 and 8 and 29 | 692 |
| 31 | limit 30 to "reviews (maximizes specificity)" | 7 |
| 32 | randomized controlled trial/ | 354278 |
| 33 | controlled clinical trial/ | 387982 |
| 34 | single blind procedure/ or double blind procedure/ | 136009 |
| 35 | crossover procedure/ | 40442 |
| 36 | random*.tw. | 920248 |
| 37 | placebo*.tw. | 208715 |
| 38 | ((singl* or doubl*) adj (blind* or mask*)).tw. | 167241 |
| 39 | (crossover or cross over or factorial* or latin square).tw. | 98352 |
| 40 | (assign* or allocat* or volunteer*).tw. | 510894 |
| 41 | 32 or 33 or 34 or 35 or 36 or 37 or 38 or 39 or 40 | 1576310 |
| 42 | 30 and 41 | 195 |
| 43 | exp cohort analysis/ | 179884 |
| 44 | exp longitudinal study/ | 70133 |
| 45 | exp prospective study/ | 264022 |
| 46 | exp follow up/ | 841502 |
| 47 | cohort$.tw. | 423779 |
| 48 | 43 or 44 or 45 or 46 or 47 | 1422519 |
| 49 | 30 and 48 | 82 |

**CINAHL**

| # | Query | Results |
| --- | --- | --- |
| 1 | (MH "Adolescence") OR (MH "Child") OR (MH "Child, Preschool") OR (MH "Infant") | 376,491 |
| 2 | TI ( child* or adolescen* or teen* or infan* or boy? or girl? or pediatric* or paediatric* ) OR AB ( child* or adolescen* or teen* or infan* or boy? or girl? or pediatric* or paediatric* ) | 268,913 |
| 3 | (MH "Vomiting") OR (MH "Diarrhea") | 5,763 |
| 4 | (MH "Gastroenteritis") OR (MH "Enteritis") | 1,274 |
| 5 | (MH "Dehydration") | 2,158 |
| 6 | TI ( diarrh* or vomit*or gastroenteritis or enteritis ) AND AB ( diarrh* or vomit*or gastroenteritis or enteritis ) | 768 |
| 7 | TI dehydrat* AND AB dehydrat* | 223 |
| 8 | 3 OR 4 OR 5 OR 6 OR 7 | 8,797 |
| 9 | 1 OR 2 | 455,043 |
| 10 | 8 AND 9 | 3,102 |
| 11 | (MH "Intravenous Therapy") OR (MH "Infusions, Intravenous") | 6,534 |
| 12 | TI ( intravenous or intra-venous or iv ) OR AB ( intravenous or intra-venous or iv ) | 29,716 |
| 13 | (MH "Fluid Therapy") OR (MH "Fluid Resuscitation") | 3,550 |
| 14 | (MH "Rehydration Solutions") | 199 |
| 15 | (MH "Sodium Chloride") | 1,348 |
| 16 | TI ( rehydrat* or hydrat* or fluid* or saline or solution* ) OR AB ( rehydrat* or hydrat* or fluid* or saline or solution* ) | 44,923 |
| 17 | 13 OR 14 OR 15 OR 16 | 47,537 |
| 18 | 11 OR 12 | 33,651 |
| 19 | 17 AND 18 | 3,546 |
| 20 | TI ( rapid* or ultrarapid* or ultra-rapid* ) OR AB ( rapid* or ultrarapid* or ultra-rapid* ) | 32,113 |
| 21 | 19 AND 20 | 244 |
| 22 | TI ( ((fast* or time? or timing or hour? or minute?) N10 (rehydrat* or hydrat* or fluid* or saline or solution*)) ) OR AB ( ((fast* or time? or timing or hour? or minute?) N10 (rehydrat* or hydrat* or fluid* or saline or solution*)) ) | 1,892 |
| 23 | 18 AND 22 | 332 |
| 24 | TI ( ((rapid* or ultrarapid* or ultra-rapid* or fast* or time? or timing or hour? or minute?) AND (rehydrat* or hydrat* or fluid* or saline or solution*)) ) OR AB ( ((rapid* or ultrarapid* or ultra-rapid* or fast* or time? or timing or hour? or minute?) N5 (rehydrat* or hydrat* or fluid* or saline or solution*)) ) | 1,472 |
| 25 | TI riv OR AB riv | 5 |
| 26 | 21 OR 23 OR 24 OR 25 | 1,748 |
| 27 | 10 AND 26 | 39 |

**GLOBAL HEALTH**

| # ▼ | Searches | Results |
| --- | --- | --- |
| 1 | children/ or boys/ or girls/ or preschool children/ or school children/ or adolescents/ or infants/ or paediatrics/ | 218603 |
| 2 | (child* or adolescen* or teen* or infan* or boy? or girl? or pediatric* or paediatric*).ti,ab. | 291302 |
| 3 | 1 or 2 | 307512 |
| 4 | gastroenteritis/ or enteritis/ or diarrhoea/ or vomiting/ | 27719 |
| 5 | (diarrh* or vomit*or gastroenteritis or enteritis).ti,ab. | 35369 |
| 6 | dehydration/ | 1163 |
| 7 | dehydrat*.ti,ab. | 5611 |
| 8 | 4 or 5 or 6 or 7 | 46401 |
| 9 | intravenous injection/ | 1349 |
| 10 | (intravenous or intra-venous or iv).ti,ab. | 49633 |
| 11 | 9 or 10 | 49733 |
| 12 | fluid therapy/ | 579 |
| 13 | rehydration/ | 401 |
| 14 | sodium chloride/ | 3427 |
| 15 | 12 or 13 or 14 | 4313 |
| 16 | 11 and 15 | 283 |
| 17 | ((intravenous or intra-venous or iv) adj10 (rehydrat* or hydrat* or fluid* or saline or solution*)).ti,ab. | 2562 |
| 18 | 16 or 17 | 2645 |
| 19 | (rapid* or ultrarapid* or ultra-rapid*).ti,ab. | 94460 |
| 20 | Time/ | 1554 |
| 21 | 19 or 20 | 95959 |
| 22 | 18 and 21 | 211 |
| 23 | ((fast* or time? or timing or hour? or minute?) adj10 (rehydrat* or hydrat* or fluid* or saline or solution*)).ti,ab. | 5686 |
| 24 | 11 and 23 | 456 |
| 25 | ((rapid* or ultrarapid* or ultra-rapid* or fast* or time? or timing or hour? or minute?) adj5 (rehydrat* or hydrat* or fluid* or saline or solution*)).ti,ab. | 3204 |
| 26 | ((rapid* or ultrarapid* or ultra-rapid* or fast* or time? or timing or hour? or minute?) and (rehydrat* or hydrat* or fluid* or saline or solution*)).ti. | 406 |
| 27 | riv.ti,ab. | 33 |
| 28 | 22 or 24 or 25 or 26 or 27 | 3878 |
| 29 | 3 and 8 and 28 | 133 |
| 30 | (random* or trial or placebo* or crossover or "cross over" or ((singl* or doubl*) adj1 (blind* or mask*)) or assign* or allocat* or volunteer*).mp. | 198398 |
| 31 | 29 and 30 | 49 |
| 32 | cohort$.tw. | 72699 |
| 33 | cohort studies/ | 2617 |
| 34 | 32 or 33 | 72699 |
| 35 | 29 and 34 | 6 |

**COCHRANE LIBRARY**

| ID | Search |
| --- | --- |
| #1 | (child* or adolescen* or teen* or infan* or boy? or girl? or pediatric* or paediatric*):ti,ab,kw (Word variations have been searched) |
| #2 | diarrh* or vomit*or gastroenteritis or enteritis:ti,ab,kw (Word variations have been searched) |
| #3 | dehydrat*:ti,ab,kw (Word variations have been searched) |
| #4 | #2 or #3 |
| #5 | MeSH descriptor: [Infusions, Intravenous] explode all trees |
| #6 | MeSH descriptor: [Administration, Intravenous] this term only |
| #7 | (intravenous or intra-venous or iv):ti,ab,kw (Word variations have been searched) |
| #8 | #5 or #6 or #7 |
| #9 | MeSH descriptor: [Fluid Therapy] explode all trees |
| #10 | MeSH descriptor: [Rehydration Solutions] explode all trees |
| #11 | MeSH descriptor: [Sodium Chloride] explode all trees |
| #12 | rehydrat* or hydrat* or fluid* or saline or solution*:ti,ab,kw (Word variations have been searched) |
| #13 | #9 or #10 or #11 or #12 |
| #14 | #8 and #13 |
| #15 | rapid* or ultrarapid* or ultra-rapid*:ti,ab,kw (Word variations have been searched) |
| #16 | MeSH descriptor: [Time Factors] explode all trees |
| #17 | #15 or #16 |
| #18 | #14 and #17 |
| #19 | ((fast* or time? or timing or hour? or minute?) near (rehydrat* or hydrat* or fluid* or saline or solution*)):ti,ab,kw (Word variations have been searched) |
| #20 | #8 and #19 |
| #21 | ((rapid* or ultrarapid* or ultra-rapid* or fast* or time? or timing or hour? or minute?) near (rehydrat* or hydrat* or fluid* or saline or solution*)):ti,ab,kw (Word variations have been searched) |
| #22 | ((rapid* or ultrarapid* or ultra-rapid* or fast* or time? or timing or hour? or minute?) and (rehydrat* or hydrat* or fluid* or saline or solution*)):ti (Word variations have been searched) |
| #23 | riv:ti,ab,kw |
| #24 | #18 or #20 or #21 or #22 or #23 |
| #25 | #1 and #4 and #24 |

**GLOBAL HEALTH LIBRARY**

| Search terms: | Hits: |
| --- | --- |
| (child OR children OR infants OR adolescents OR adolescents OR teen OR teens OR teenagers OR pediatric OR pediatrics OR paediatrics OR paediatric) AND (rapid OR rapidly OR ultrarapid OR ultra-rapid) AND (intravenous OR IV) AND (diarrhoea OR diarrhea OR vomit OR vomiting OR gastroenteritis OR enteritis OR dehydration OR dehydrate OR dehydrated) | 12 |
| (child OR children OR infants OR adolescents OR adolescents OR teen OR teens OR teenagers OR pediatric OR pediatrics OR paediatrics OR paediatric) AND (rapid OR rapidly OR ultrarapid OR ultra-rapid) AND (rehydrate OR rehydration OR rehydrated OR hydration OR hydrate OR hydrated OR fluid OR fluids OR saline) AND (diarrhoea OR diarrhea OR vomit OR vomiting OR gastroenteritis OR enteritis OR dehydration OR dehydrate OR dehydrated) | 22 |
| (child OR children OR infants OR adolescents OR adolescents OR teen OR teens OR teenagers OR pediatric OR pediatrics OR paediatrics OR paediatric) AND (fast OR hour OR hours OR minute OR minutes) AND (intravenous OR IV) AND (diarrhoea OR diarrhea OR vomit OR vomiting OR gastroenteritis OR enteritis OR dehydration OR dehydrate OR dehydrated) | 44 |
| (child OR children OR infants OR adolescents OR adolescents OR teen OR teens OR teenagers OR pediatric OR pediatrics OR paediatrics OR paediatric) AND (fast OR hour OR hours OR minute OR minutes) AND (rehydrate OR rehydration OR rehydrated OR hydration OR hydrate OR hydrated OR fluid OR fluids OR saline) AND (diarrhoea OR diarrhea OR vomit OR vomiting OR gastroenteritis OR enteritis OR dehydration OR dehydrate OR dehydrated) | 64 |

**WOK (Science Citation Index Expanded (SCI-EXPANDED); and Conference Proceedings Citation Index-Science (CPCI-S) (Web of Science)**

| Set | Results | Save search history and/or create an alertOpen a saved search history |
| --- | --- | --- |
|  |  |  |
| 1 | 1,312,847 | TOPIC: (child* or adolescen* or teen* or infan* or boy? or girl? or pediatric* or paediatric*) |
| 2 | 146,073 | TOPIC: (diarrh* or vomit*or gastroenteritis or enteritis) OR TOPIC: (dehydrat*) |
| 3 | 476,333 | TS=(intravenous or intra-venous or iv) |
| 4 | 3,878 | TOPIC: (intravenous or intra-venous or iv) AND TOPIC: (rehydrat* or hydrat* or fluid* or saline or solution*) AND TOPIC: (rapid* or ultrarapid* or ultra-rapid*) |
| 5 | 634,325 | TOPIC: ((fast* or time? or timing or hour? or minute?) SAME (rehydrat* or hydrat* or fluid* or saline or solution*)) |
| 6 | 16,425 | 5 AND 3 |
| 7 | 107,741 | TS=((rapid* or ultrarapid* or ultra-rapid* or fast* or time? or timing or hour? or minute?) NEAR/5 (rehydrat* or hydrat* or fluid* or saline or solution*)) |
| 8 | 16,665 | TI=((rapid* or ultrarapid* or ultra-rapid* or fast* or time? or timing or hour? or minute?) and (rehydrat* or hydrat* or fluid* or saline or solution*)) |
| 9 | 129,415 | 8 OR 7 OR 6 OR 4 |
| 10 | 268 | 9 AND 2 AND 1 |
| 11 | 2,295,958 | TS=((random* or trial or placebo* or crossover or "cross over" or ((singl* or doubl*) NEAR/1 (blind* or mask*)) or assign* or allocat* or volunteer*)) |
| 12 | 123 | 11 AND 10 |
| 13 | 307,644 | TS=cohort* |
| 14 | 17 | 13 AND 10 |

**Supplementary Table 2: SEARCH RESULTS (2014 to May 2017)**

MEDLINE

| # ▼ | Searches | Results |
| --- | --- | --- |
| 1 | adolescent/ or exp child/ or infant/ or infant, newborn/ | 3258052 |
| 2 | (child* or adolescen* or teen* or infan* or boy? or girl? or pediatric* or paediatric*).ti,ab. | 1804186 |
| 3 | 1 or 2 | 3687601 |
| 4 | exp diarrhea/ or vomiting/ or gastroenteritis/ or enteritis/ | 87996 |
| 5 | (diarrh* or vomit*or gastroenteritis or enteritis).ti,ab. | 104631 |
| 6 | Dehydration/ | 12156 |
| 7 | dehydrat*.ti,ab. | 37257 |
| 8 | 4 or 5 or 6 or 7 | 189786 |
| 9 | administration, intravenous/ or infusions, intravenous/ | 58252 |
| 10 | (intravenous or intra-venous or iv).ti,ab. | 589392 |
| 11 | 9 or 10 | 614195 |
| 12 | exp Fluid Therapy/ | 18032 |
| 13 | Rehydration Solutions/ | 1372 |
| 14 | Sodium Chloride/ | 56142 |
| 15 | 12 or 13 or 14 | 73909 |
| 16 | 11 and 15 | 6214 |
| 17 | ((intravenous or intra-venous or iv) adj10 (rehydrat* or hydrat* or fluid* or saline or solution*)).ti,ab. | 25183 |
| 18 | 16 or 17 | 28013 |
| 19 | (rapid* or ultrarapid* or ultra-rapid*).ti,ab. | 831899 |
| 20 | Time Factors/ | 1113891 |
| 21 | 19 or 20 | 1879955 |
| 22 | 18 and 21 | 4400 |
| 23 | ((fast* or time? or timing or hour? or minute?) adj10 (rehydrat* or hydrat* or fluid* or saline or solution*)).ti,ab. | 68715 |
| 24 | 11 and 23 | 7173 |
| 25 | ((rapid* or ultrarapid* or ultra-rapid* or fast* or time? or timing or hour? or minute?) adj5 (rehydrat* or hydrat* or fluid* or saline or solution*)).ti,ab. | 39428 |
| 26 | ((rapid* or ultrarapid* or ultra-rapid* or fast* or time? or timing or hour? or minute?) and (rehydrat* or hydrat* or fluid* or saline or solution*)).ti. | 4941 |
| 27 | riv.ti,ab. | 159 |
| 28 | 22 or 24 or 25 or 26 or 27 | 48857 |
| 29 | 3 and 8 and 28 | 557 |
| 30 | randomized controlled trial.pt. | 462409 |
| 31 | controlled clinical trial.pt. | 94057 |
| 32 | randomized.ab. | 403676 |
| 33 | placebo.ab. | 188903 |
| 34 | drug therapy.fs. | 1992751 |
| 35 | randomly.ab. | 280393 |
| 36 | trial.ab. | 422695 |
| 37 | groups.ab. | 1727320 |
| 38 | 30 or 31 or 32 or 33 or 34 or 35 or 36 or 37 | 4101978 |
| 39 | exp animals/ not humans.sh. | 4398299 |
| 40 | 38 not 39 | 3547013 |
| 41 | 29 and 40 | 268 |
| 42 | limit 29 to "reviews (maximizes specificity)" | 21 |
| 43 | 29 not (41 or 42) | 286 |
| 44 | (2014* or 2015* or 2016* or 2017*).ed,yr,dp. | 4612013 |
| 45 | 41 and 44 | 29 |
| 46 | 42 and 44 | 7 |
| 47 | 43 and 44 | 35 |

EMBASE

| # ▼ | Searches | Results |
| --- | --- | --- |
| 1 | child/ or boy/ or girl/ or infant/ or preschool child/ or school child/ or adolescent/ | 2730185 |
| 2 | (child* or adolescen* or teen* or infan* or boy? or girl? or pediatric* or paediatric*).ti,ab. | 2157527 |
| 3 | 1 or 2 | 3405712 |
| 4 | diarrhea/ or acute diarrhea/ or infantile diarrhea/ or vomiting/ or gastroenteritis/ or enteritis/ or acute gastroenteritis/ or gastroenteritis/ or infantile gastroenteritis/ | 366241 |
| 5 | (diarrh* or vomit*or gastroenteritis or enteritis).ti,ab. | 136138 |
| 6 | dehydration/ | 33360 |
| 7 | dehydrat*.ti,ab. | 43705 |
| 8 | 4 or 5 or 6 or 7 | 456546 |
| 9 | intravenous drug administration/ | 364893 |
| 10 | (intravenous or intra-venous or iv).ti,ab. | 762180 |
| 11 | 9 or 10 | 1007389 |
| 12 | fluid therapy/ | 17895 |
| 13 | rehydration/ | 5452 |
| 14 | 12 or 13 | 23119 |
| 15 | 11 and 14 | 3618 |
| 16 | sodium chloride/iv | 1506 |
| 17 | infusion fluid/ | 10739 |
| 18 | ((intravenous or intra-venous or iv) adj10 (rehydrat* or hydrat* or fluid* or saline or solution*)).ti,ab. | 33382 |
| 19 | 15 or 16 or 17 or 18 | 43068 |
| 20 | (rapid* or ultrarapid* or ultra-rapid*).ti,ab. | 973312 |
| 21 | Time/ | 389168 |
| 22 | 20 or 21 | 1345886 |
| 23 | 19 and 22 | 3919 |
| 24 | ((fast* or time? or timing or hour? or minute?) adj10 (rehydrat* or hydrat* or fluid* or saline or solution*)).ti,ab. | 83940 |
| 25 | 11 and 24 | 10248 |
| 26 | ((rapid* or ultrarapid* or ultra-rapid* or fast* or time? or timing or hour? or minute?) adj5 (rehydrat* or hydrat* or fluid* or saline or solution*)).ti,ab. | 47592 |
| 27 | ((rapid* or ultrarapid* or ultra-rapid* or fast* or time? or timing or hour? or minute?) and (rehydrat* or hydrat* or fluid* or saline or solution*)).ti. | 5121 |
| 28 | riv.ti,ab. | 234 |
| 29 | 23 or 25 or 26 or 27 or 28 | 58335 |
| 30 | 3 and 8 and 29 | 816 |
| 31 | limit 30 to "reviews (maximizes specificity)" | 12 |
| 32 | randomized controlled trial/ | 445616 |
| 33 | controlled clinical trial/ | 430203 |
| 34 | single blind procedure/ or double blind procedure/ | 162532 |
| 35 | crossover procedure/ | 50870 |
| 36 | random*.tw. | 1178683 |
| 37 | placebo*.tw. | 252743 |
| 38 | ((singl* or doubl*) adj (blind* or mask*)).tw. | 198243 |
| 39 | (crossover or cross over or factorial* or latin square).tw. | 118939 |
| 40 | (assign* or allocat* or volunteer*).tw. | 628735 |
| 41 | 32 or 33 or 34 or 35 or 36 or 37 or 38 or 39 or 40 | 1957248 |
| 42 | 30 and 41 | 225 |
| 43 | 30 not (31 or 42) | 587 |
| 44 | (2014* or 2015* or 2016* or 2017*).dc,yr,dp. | 5671476 |
| 45 | 31 and 44 | 4 |
| 46 | 42 and 44 | 38 |
| 47 | 43 and 44 | 130 |

GLOBAL HEALTH

| **# ▼** | **Searches** | **Results** |
| --- | --- | --- |
| 1 | children/ or boys/ or girls/ or preschool children/ or school children/ or adolescents/ or infants/ or paediatrics/ | 268812 |
| 2 | (child* or adolescen* or teen* or infan* or boy? or girl? or pediatric* or paediatric*).ti,ab. | 350762 |
| 3 | 1 or 2 | 370523 |
| 4 | gastroenteritis/ or enteritis/ or diarrhoea/ or vomiting/ | 33575 |
| 5 | (diarrh* or vomit*or gastroenteritis or enteritis).ti,ab. | 41351 |
| 6 | dehydration/ | 1622 |
| 7 | dehydrat*.ti,ab. | 6903 |
| 8 | 4 or 5 or 6 or 7 | 55051 |
| 9 | intravenous injection/ | 1884 |
| 10 | (intravenous or intra-venous or iv).ti,ab. | 57973 |
| 11 | 9 or 10 | 58110 |
| 12 | fluid therapy/ | 759 |
| 13 | rehydration/ | 532 |
| 14 | sodium chloride/ | 4685 |
| 15 | 12 or 13 or 14 | 5863 |
| 16 | 11 and 15 | 369 |
| 17 | ((intravenous or intra-venous or iv) adj10 (rehydrat* or hydrat* or fluid* or saline or solution*)).ti,ab. | 2964 |
| 18 | 16 or 17 | 3073 |
| 19 | (rapid* or ultrarapid* or ultra-rapid*).ti,ab. | 114147 |
| 20 | Time/ | 2092 |
| 21 | 19 or 20 | 116164 |
| 22 | 18 and 21 | 231 |
| 23 | ((fast* or time? or timing or hour? or minute?) adj10 (rehydrat* or hydrat* or fluid* or saline or solution*)).ti,ab. | 6993 |
| 24 | 11 and 23 | 542 |
| 25 | ((rapid* or ultrarapid* or ultra-rapid* or fast* or time? or timing or hour? or minute?) adj5 (rehydrat* or hydrat* or fluid* or saline or solution*)).ti,ab. | 3889 |
| 26 | ((rapid* or ultrarapid* or ultra-rapid* or fast* or time? or timing or hour? or minute?) and (rehydrat* or hydrat* or fluid* or saline or solution*)).ti. | 496 |
| 27 | riv.ti,ab. | 36 |
| 28 | 22 or 24 or 25 or 26 or 27 | 4670 |
| 29 | 3 and 8 and 28 | 143 |
| 30 | (random* or trial or placebo* or crossover or "cross over" or ((singl* or doubl*) adj1 (blind* or mask*)) or assign* or allocat* or volunteer*).mp. | 248063 |
| 31 | 29 and 30 | 55 |
| 32 | 29 not 31 | 88 |
| 33 | (2014* or 2015* or 2016* or 2017*).yr,dp. | 559014 |
| 34 | 31 and 33 | 6 |
| 35 | 32 and 33 | 6 |

GLOBAL HEALTH LIBRARY

| **Search terms:** | **Hits:** |
| --- | --- |
| (child OR children OR infants OR adolescents OR adolescents OR teen OR teens OR teenagers OR pediatric OR pediatrics OR paediatrics OR paediatric) AND (rapid OR rapidly OR ultrarapid OR ultra-rapid) AND (intravenous OR IV) AND (diarrhoea OR diarrhea OR vomit OR vomiting OR gastroenteritis OR enteritis OR dehydration OR dehydrate OR dehydrated) | 6 |
| (child OR children OR infants OR adolescents OR adolescents OR teen OR teens OR teenagers OR pediatric OR pediatrics OR paediatrics OR paediatric) AND (rapid OR rapidly OR ultrarapid OR ultra-rapid) AND (rehydrate OR rehydration OR rehydrated OR hydration OR hydrate OR hydrated OR fluid OR fluids OR saline) AND (diarrhoea OR diarrhea OR vomit OR vomiting OR gastroenteritis OR enteritis OR dehydration OR dehydrate OR dehydrated) | 6 |
| (child OR children OR infants OR adolescents OR adolescents OR teen OR teens OR teenagers OR pediatric OR pediatrics OR paediatrics OR paediatric) AND (fast OR hour OR hours OR minute OR minutes) AND (intravenous OR IV) AND (diarrhoea OR diarrhea OR vomit OR vomiting OR gastroenteritis OR enteritis OR dehydration OR dehydrate OR dehydrated) | 10 |
| (child OR children OR infants OR adolescents OR adolescents OR teen OR teens OR teenagers OR pediatric OR pediatrics OR paediatrics OR paediatric) AND (fast OR hour OR hours OR minute OR minutes) AND (rehydrate OR rehydration OR rehydrated OR hydration OR hydrate OR hydrated OR fluid OR fluids OR saline) AND (diarrhoea OR diarrhea OR vomit OR vomiting OR gastroenteritis OR enteritis OR dehydration OR dehydrate OR dehydrated) | 17 |
|  | 39 |

SUMMARY TABLE FOR UPDATED SEARCH (2014 – May 2017)

| **Database:** | **Interface:** | **Coverage:** | **Dates:** | **Reviews:** | **Trials:** | **Cohort & Other:** | **Total:** |
| --- | --- | --- | --- | --- | --- | --- | --- |
| CINAHL | EBSCOHost | 1982-present | 11/05/17 | 0 | 0 | 9 |  |
| Cochrane Database of Systematic Reviews | Cochrane Library, Wiley | Issue 5 of 12, May 2017 | 11/05/17 | 3 | 0 | 0 |  |
| Cochrane Central Register of Controlled Trials | Cochrane Library, Wiley | Issue 4 of 12, April 2017 | 11/05/17 | 0 | 20 | 0 |  |
| Database of Abstracts of Reviews of Effects | Cochrane Library, Wiley | Issue 2 of 4, April 2015 | 11/05/17 | 0 | 0 | 0 |  |
| Embase | OvidSP | 1974 to 2017 May 10 | 11/05/17 | 4 | 38 | 130 |  |
| Global Health | OvidSP | 1973 to 2017 Week 17 | 11/05/17 | 0 | 6 | 6 |  |
| Global Health Library - regional indexes | <http://www.globalhealthlibrary.net/php/index.php> | | 11/05/17 | 0 | 0 | 39 |  |
| Ovid MEDLINE(R) Epub Ahead of Print, In-Process & Other Non-Indexed Citations, Ovid MEDLINE(R) Daily and Ovid MEDLINE(R) | OvidSP | 1946 to Present | 11/05/17 | 7 | 29 | 35 |  |
| Science Citation Index, Social Science Citation Index & Conference Proceedings Citation Index-Science | Web of Science Core Collection, Thomson Reuters | 1945-present | 11/05/17 | 0 | 15 | 33 |  |
| Total: |  |  |  | 14 | 108 | 252 | 374 |
| Duplicates excluded: |  |  |  |  |  |  | 139 |
| **Final Total:** |  |  |  | 5 | 45 | 185 | 235 |
|  |  |  |  |  |  |  |  |
| **Notes:** |  |  |  |  |  |  |  |
| References de-duplicated against results of original search held in an Endnote database |  |  |  |  |  |  |  |
| Searches restricted to articles published and/or added to databases since 2014 - as a result a few early papers included |  |  |  |  |  |  |  |

**Supplementary Table 2: EXCLUDED STUDIES**

| **Author, year** | **Comparison** |
| --- | --- |
| Allen, 2014^a^ | Plasma-Lyte A (PLA) to 0.9% normal saline (NS) |
| Levy JA, 2013^b^ | IV dextrose vs. 0.9% saline |
| Mahajan V, 2012^c^ | Ringer’s lactate vs. 0.9% saline |
| Neville KA, 2006^d^ | 0.9% saline + 2.5% dextrose (NS) or 0.45% saline + 2.5% dextrose |
| Spandorfer PR, 2005^e^ | IV vs. oral rehydration |
| Juca CA, 2005^f^ | Polyelectrolyte vs. 0.9% saline |
| Nager AL, 2002^g^ | Rapid nasogastric hydration vs. rapid intravenous hydration |
| Atherley-John YC, 2002^h^ | IV vs. oral rehydration |
| Mackenzie A, 1991^i^ | IV vs. oral rehydration |
| Rahman O, 1988^j^ | Rapid intravenous rehydration with a single polyelectrolyte solution with or without dextrose |
| Vesikari T, 1987^k^ | IV vs. oral rehydration |
| Listernick R, 1986^l^ | IV vs. oral rehydration |
| Sharifi J, 1985^m^ | IV vs. oral rehydration |
| Kartha GB, | Ringer’s lactate vs. 0.9% saline |
| Allen CH, 2016 | Plasma-Lyte vs. 0.9% saline |

1. Allen C, Goldman R, Simon H, et al. Balanced crystalloid or saline in pediatric gastroenteritis: A randomized controlled trial. In: Proceedings of the 2014 Annual Meeting of the Society for Academic Emergency Medicine, SAEM 2014 Dallas, TX United States. Acad Emerg Med 2014; 21 (5 SUPPL. 1):S196-7.
2. Levy J, Bachur R, Monuteaux M, et al. Intravenous dextrose for children with gastroenteritis and dehydration: a double-blind randomized controlled trial. Ann of Emer Med 2013;61(3):281-8.
3. Mahajan V, Sajan S, Sharma A, et al. Ringers lactate vs Normal saline for children with acute diarrhea and severe dehydration- a double blind randomized controlled trial. Indian Pediatr. 2012;49(12):963-8.
4. Neville K, Verge C, Rosenberg A, et al. Isotonic is better than hypotonic saline for intravenous rehydration of children with gastroenteritis: a prospective randomised study. Arch Dis Child 2006;91(3):226-32.
5. Spandorfer P, Alessandrini E, Joffe M, et al. Oral versus intravenous rehydration of moderately dehydrated children: a randomized, controlled trial. Pediatrics 2005;115(2):295-301.
6. Juca C, Rey L, Martins C. Comparison between normal saline and a polyelectrolyte solution for fluid resuscitation in severely dehydrated infants with acute diarrhoea. Ann Trop Paediatr 2005;**25**(4):253-60.
7. Nager A, Wang V. Comparison of nasogastric and intravenous methods of rehydration in pediatric patients with acute dehydration. Pediatrics 2002;**109**(4):566-72.
8. Atherly-John Y, Cunningham S, Crain E. A randomized trial of oral vs intravenous rehydration in a Pediatric Emergency Department. Archives of Pediatrics and Adolescent Medicine 2002;**156**(12):1240-3.
9. Mackenzie A, Barnes G. Randomised controlled trial comparing oral and intravenous rehydration therapy in children with diarrhoea. BMJ 1991; 303(6799):393-6.
10. Rahman O, Bennish ML, Alam AN, Salam MA. Rapid intravenous rehydration by means of a single polyelectrolyte solution with or without dextrose. J Pediatr 1988;**113**(4):654-60.
11. Vesikari T, Isolauri E, Baer M. A comparative trial of rapid oral and intravenous rehydration in acute diarrhoea. Acta Paediatrica Scandinavica 1987;76(2):300-5.
12. Listernick R, Zieserl E, Davis A. Outpatient oral rehydration in the United States. Am J of Dis Child 1986;140(3):211-5.
13. Sharifi J, Ghavami F, Nowrouzi Z, et al. Oral versus intravenous rehydration therapy in severe gastroenteritis. Arch Dis Child1985;60(9):856-60.

n) Gayathri Bhuvaneswaran Kartha, Ramachandran Rameshkumar, Subramanian Mahadevan. Randomized Double-Blind Trial of Ringer's Lactate versus Normal Saline in Pediatric Acute Severe Diarrheal Dehydration. *Journal of Pediatric Gastroenterology & Nutrition.* 2017. 18:18

o) Coburn H. Allen, Ran D. Goldman, Seema Bhatt, Harold K. Simon, Marc H. Gorelick, Philip R. Spandorfer, David M. Spiro, Sharon E. Mace, David W. Johnson, Eric A. Higginbotham, Hongyan Du, Brendan J. Smyth, Carol R. Schermer, Stuart L. Goldstein. A randomized trial of Plasma-Lyte A and 0.9 % sodium chloride in acute pediatric gastroenteritis. *BMC Pediatrics.* 2016. 16:117

**Supplementary Table 3: RISK OF BIAS FOR INCLUDED STUDIES**

**Freedman 2011**

| **Bias** | **Author’s judgement** | **Support for judgement** |
| --- | --- | --- |
| Allocation concealment (selection bias) | Low | Computer generated permuted block randomisation sequence; stratification by severity of dehydration.  Allocation was concealed using of sequentially numbered sealed opaque envelopes which were provided to the research nurse only after consent was obtained and thereafter opened sequentially after information on the participant was written on the appropriate envelope. The randomisation code remained secured until enrolment and data entry were complete. |
| Blinding of participants and personnel (performance bias) | Low | The research nurse and participants were blinded to treatment allocation. Opaque bags were used to conceal the infusion bags and tubing, and soundproof boxes used to conceal the infusion pumps. The bedside nurse who was unblinded to set the intravenous rate was not allowed to communicate any information about the infusion or the child’s clinical status |
| Blinding of assessors (detection bias) | Low | The attending physician who performed participant assessments and made management decisions was blinded to the treatment groups |
| Incomplete outcome data(attrition bias) | Low | Results were reported on an intention to treat basis and included all participants in each groups |
| Selective reporting (reporting bias) | Low | The trial was registered at ClinicalTrials.gov and there does not appear to be any deviations in study conduct or outcomes pre-specified in the study protocol and the publication. |
| Other | Low | The study sponsors played no role in the study design or data collection, analysis, and interpretation or in the wiring of the article.  No other apparent risk of bias. |

**Nager 2008**

| **Bias** | **Author’s judgement** | **Support for judgement** |
| --- | --- | --- |
| Allocation concealment (selection bias) | Unclear | Computer generated random number scheme was used however information on allocation concealment was not provided |
| Blinding of participants and personnel (performance bias) | Unclear | The authors report that the investigator performing the follow up telephone call was blinded to treatment allocation. However information was not provided on blinding of participants and nursing staff that did the monitoring during the admission period. |
| Blinding of assessors (detection bias) | Low | One of the investigators who contacted caregivers by telephone after discharge was blinded to treatment group. |
| Incomplete outcome data (attrition bias) | High | ‘Ninety-one percent of all study participants (89% ultra and 93% standard) completed a standardised questionnaire 24 hours after discharge’. The reasons for non-completion of questionnaires were not provided. The method of data analysis was not provided therefore it is not clear if all participants were included in the analysis.  (b) Information regarding the use of standardised discharge criteria, which was one of the specified methods for assessment of rehydration effectiveness, is unclear and data relating to this outcome measure have not been reported.  (c) The authors have not pre-specified a method of analysis. However, they excluded any patient requiring admission to  hospital from their analysis and the basis for this is unclear.  (d) One of the study objectives was to compare data including success and timing of rehydration and the number of patients who were admitted but have excluded patients who required admission to hospital from their analysis. |
| Selective reporting (reporting bias) | Unclear | There is no published protocol for this study in the public domain for this study |
| Other | High | (a) Possible intention bias: The trialists set out to prove a pre-conceived belief that ultrarapid rehydration could be performed effectively with similar results as standard rehydration therapy which raises concerns regarding clinical equipoise.  (b) Study design: This was a pilot study with very low power (60%) and unclear criteria for assessment of equivalence.  (c) Selection bias: The use of very strict exclusion criteria - e.g. exclusion of children with severe dehydration and those with significant laboratory abnormalities on initial bedside testing could potentially bias the results and certainly makes the findings of this study not generalisable to routine clinical practice.  (d) Study objectives are unclear, and the outcome measures not robust methods of assessing the main study objective.  (e) The authors have drawn a conclusion of comparable efficacy but results were non-significant.  (f) Method of reporting results not transparent and not in accordance with CONSORT guidelines. |

**Azarfar 2014**

| **Bias** | **Author’s judgement** | **Support for judgement** |
| --- | --- | --- |
| Allocation concealment (selection bias) | Unclear | ‘Random sequence was generated by the statistical adviser of the research program using a computerised randomiser’.  However, information on allocation concealment was not provided. |
| Blinding (performance bias) | Unclear | Information not provided |
| Blinding of assessors (detection bias) | Unclear | Information not provided |
| Incomplete data (attrition bias) | High | (a)The authors have provided results of their primary outcome however; they have not clearly provided information on outcomes assessed on day 3 of admission for those admitted.  (b) Nine participants from the intervention group were excluded from the study. The number of participants excluded from the control group is not reported therefore it is unclear whether this was balanced between both groups  (c)The number of participants for which follow up information was obtained is unclear. |
| Selective reporting (reporting bias) | High | (a) The primary and secondary outcome measures stated in the study protocol differ from what is published. The authors have reported their secondary outcomes (as per their protocol) as their primary outcomes in the publication, and vice versa.  (b)The intended sample size stated in the protocol (n=70; 35 participants in each group) is different to what has been published and the authors have not indicated reasons for over recruitment.  (c) The authors have not specified what type of analysis was performed and whether results from the nine excluded participants in the intervention group were included in their analysis. |
| Other | High | (a) Small sample size.  (b) No power calculation  (c) Method of reporting results not transparent and not in accordance with CONSORT guidelines. |
